# Method of Data Selection for Turning of Inconel 718 Alloy Obtained by Casting and Laser Sintering Powder

**DOI:** 10.3390/ma15041448

**Published:** 2022-02-15

**Authors:** Ksenia Latosińska, Grzegorz Struzikiewicz, Wojciech Zębala

**Affiliations:** 1 Chair of Production Engineering, Faculty of Mechanical Engineering, Cracow University of Technology, 31-864 Cracow, Poland; wojciech.zebala@pk.edu.pl; 2 Chair of Manufacturing Systems, Faculty of Mechanical Engineering and Robotics, AGH University of Science and Technology, 30-059 Cracow, Poland; gstruzik@agh.edu.pl

**Keywords:** turning, DMLS, Inconel 718, specific cutting force, borazon

## Abstract

This article focuses on the issues related to the machining of DMLS (Direct Metal Laser Sintering) laser sintered parts made of Inconel 718 alloy. Longitudinal turning with CBN (cubic boron nitride) tool inserts is analyzed. The authors made an attempt to establish a procedure to find the optimal finishing cutting parameters while minimizing the specific cutting force and taking into account the machined surface quality criterion. During experiments the influence of cutting data on the values of cutting force and specific cutting force were performed. Moreover, the results of measurements of surface roughness parameters and the results of analysis of chip form are presented as well. Cast Inconel 718 has also been tested for comparative purposes. The variability of the material’s hardening state during machining was found, as well as the variability of the specific cutting force value as a function of the cross-sectional shape of the cutting layer. The values of all components of the total cutting force for turning the material obtained by the additive method are lower than for turning the cast material by approximately 32%. At the end of the article, the authors present an application of the proposed optimization algorithm. It was established that by changing the cross-section shape of the cutting layer, it was possible to perform the turning process at a specific cutting force value of 22% less, which is achieved by reducing the cross-section size.

## 1. Introduction

Nickel-cobalt-based alloys, and Inconel 718 in particular, are one of the most widely used superalloys in the aerospace and energy industries today. They are used in the production of aircraft engine parts and in the energy industry due to their unique properties such as resistance to high temperatures, good thermal fatigue properties, corrosion resistance, and good mechanical properties in extreme conditions [1,2]. These materials constitute about 50% of the components of the gas aircraft engine due to their unique properties [3,4]. Characteristic features of alloys based on nickel and cobalt include high hardness, a tendency to react with the tool (the formation of built-up edges on the blade), high strength at high temperature, low thermal conductivity, the ability to strengthen in the cutting zone and, as a result, the hardening of the alloy in the top layer [5,6]. When processing these type of materials, special attention is paid to the quality of the surface and the cohesion of the surface layer of the parts, as it affects their durability, functionality and fatigue strength. Optimization of the treatment of such materials is considered in terms of surface roughness, maximization of the cutting edge service life, and minimization of production costs [7,8].

The main methods of forming the Inconel 718 alloy are forging, casting and sintering [9]; thanks to the sintering technology, both raw material and energy can be saved; furthermore, the sintered elements are characterized by good mechanical properties and precision [10]. The production of materials using the additive manufacturing method (AM) consists of the production of objects based on the execution of a three-dimensional computer model (3D CAD) followed by the application of accumulated layers of material using various methods. AM technologies allow for the production of very complex and intricate shapes with relatively high accuracy and a high degree of structure densification [11,12]. This method allows us to shorten the production time of materials and reduce their costs [13] while maintaining their characteristic features [14,15]. The DMLS method (Direct Metal Laser Sintering) is an incremental method that melts successive layers of metal powder with a laser beam in an inert gas atmosphere which is filled with the sintering chamber in order to minimize the oxidation process of the metal powder [16]. Inconel 718 is a complex material intended for machining in AM processes because it contains a large number of alloying elements and is a precipitation strengthened alloy [17,18]. The structure and microstructure of alloys made of metal powders is different than that of cast alloys [19]. There are residual stresses that can be mitigated by various in-process methods such as heating, process planning, feedback control and post-processing (e.g., machining and heat treatment) [20]. The authors are trying to improve the sintering process of nickel-based alloys by modifying parameters such as pressure, temperature and the electric field [21].

Inconel 718 belongs to the group of difficult-to-cut materials (superalloys). Thakur et al. [22] described the influence of cutting parameters and tool wear on the surface condition of the material after machining. They noticed that the surface roughness is closely related to changes in the structure as a result of high temperatures in the machining zone (grain growth) and mechanical strains (strain hardening and DRX). They demonstrated that most surface defects appearing during machining (deformed grains, crack-king, debris, feed marks, carbide particles, surface cavities, adhered material particles, surface plucking, slip zones, and redeposited materials) are the result of improper selection of machining parameters and excessive tool wear. Residual stress studies and simulations for the treatment of Inconel 718 have already been carried out in many previous studies. The residual stresses along with the cutting parameters are influenced by the hardening speed (cold work) and the yield point resulting from the preload [23,24]. The trends of these changes differ depending on the increase in feed, depth of cut and cutting speed [25,26]. Many authors have studied the influence of various machining parameters on the obtained final results of the experiments, and they have shown the following dependencies.

Hua et al. [27] investigated the influence of the direction and magnitude of the main residual stress on the fatigue strength of the turned Inconel 718 alloy. They showed that the main residual stress is much greater than the residual surface stress and that it increases with increasing feed. They found that the main residual stresses had the greatest influence on the fatigue properties of the turned alloy. Peng et al. [28] examined the residual stresses on the surface of the turned Inconel 718 alloy. They showed that with the increase of the feed, the cutting forces increased regardless of the value of the cutting speed, and simultaneously, with the increase of the depth of cut, the cutting forces increased, but at high cutting speeds they were lower than with low cutting speeds. Hua et al. [29] investigated the effect of cutting speed, feed rate and nose radius on roughness, microhardness and the degree of work hardening of Inconel 718. They showed that an increase in the feed rate generated greater cutting force and a greater degree of material compaction. Cutting speed did not significantly affect the surface roughness as opposed to the feed rate and the nose radius of the cutting edge. The use of larger corner radii reduced the degree of work hardening and the fatigue crack propagation threshold. ČEP et al. [30] checked the influence of cutting parameters as well as manufacturing technology on the surface structure of the material after the turning process. Other authors have also studied the effect of cutting speed and other machining conditions on the surface condition after turning for nickel-based alloys made with various manufacturing technologies. Numerous authors focus on studying the effects of parameters such as feed, cutting speed, tools on the microhardness and roughness of the machined surface in the turning process [31,32].

Currently, there are no dedicated procedures for optimizing the machining of sintered materials, including the sintered Inconel 718 alloy. The authors made an attempt to establish a procedure to find the optimal finishing cutting parameters for laser sintered powder nickel-cobalt alloy while minimizing the cutting resistance and taking into account the machined surface quality criterion. An analysis of the machining of the longitudinal turning of a part made of cast and sintered Inconel 718 was carried out.

The article is organized as follows: Section 1 provides an introduction. The tests and analysis are presented in Section 2 (materials and experiments), while Section 3 describes the algorithm for the turning process of the Inconel 718 alloy and shows the application of the created algorithm. Section 4 presents the conclusions.

## 2. Materials and Experiments

As part of the study of the finishing process of laser sintered powder Inconel 718, an attempt was made to determine the effect of cutting parameters on the specific cutting resistance. Cast Inconel 718 was also tested as a comparison.

DMLS samples were made using a Renishaw AM 250 (Renishaw, Wotton-under-Edge, UK). The following parameters were used to prepare the samples: power 185 W, speed 289 mm/s, exposure time 100 µs, distance between sintering points 65 µm, distance between transition lines 0.13 mm, layer thickness 50 µm. The mechanical properties of the material and the chemical composition are presented in Table 1 and Table 2.

While testing, the components of the cutting forces, the roughness of the machined surface and the form of chips obtained in the process were measured. A Talysurf Intra 50 profilographometer by Taylor Hobson (Leicester, UK) was used for the measurements. Microscopic analysis of the machined surface was performed using a microscope by Bresser (Advance ICD, Bresser, Rhede, Germany). In order to record and analyze the components of the cutting forces, a measuring track was used, built on the piezoelectric dynamometer 9257B and amplifier 5070B by Kistler (Winterthur, Switzerland). The measurement of the maximum temperature value in the machined zone was carried out with the use of the SC620 thermal imaging camera and the ThermaCam Researcher program by Flir Systems (Wilsonville, OR, USA).

The SDJCR1616H11 holder from MMC Hardmetal (Group Company of Mitsubishi Materials Corporation) (Tamworth, UK) with cutting inserts made of BN2000 CBN from Sumitomo (Tokyo, Japan) was applied. The cutting parameter values are within the range recommended by the tool manufacturer.

Based on the adopted research plan, the components of the total cutting force during longitudinal turning of cast and printed Inconel 718 alloy were measured. The influence of the cross-section of the cutting layer *A_D_* on the values of the components of the total cutting force, i.e., main *F_c_*, feed *F_f_* and back *F_p_*, as well as specific cutting force, was analyzed. The cross-section of the cutting layer was a function of variable cutting parameters, i.e., feed *f* and depth of cut *a_p_*. All trials and measurements were conducted three to five times.

Specific cutting force *k_c_* is defined as the ratio of the main cutting force *F_c_* to the cross-section area of the cutting layer *A_D_* (1).

(1) $$k_{c}=\frac{F_{c}}{A_{D}}=\frac{F_{c}}{f\cdot a_{p}}\;[N/{\mathrm{mm}}^{2}]$$

**Figure 1:**
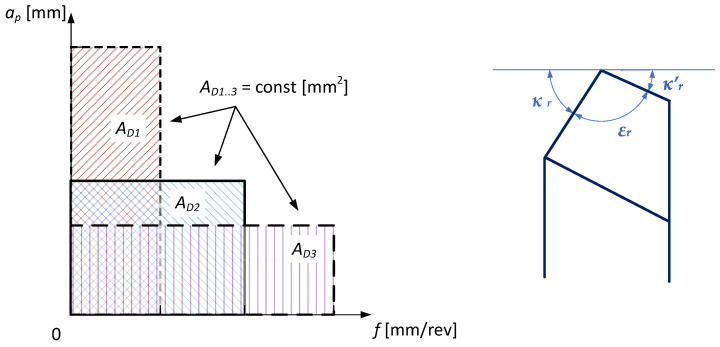
A simplified diagram of the cross-section shapes of the cutting layer while maintaining a constant cross-section for turning with entering angle *K_R_* = 90°.

For materials of different groups (with a different chemical composition and structure) with the same tensile strength, the specific cutting force may change. Inconel 718 is a specific example because it hardens upon treatment. The value of the specific cutting force depends on the value of the cross-section *A_D_*. In order to determine the influence of the shape of the cross-section of the cutting layer on the value of the specific cutting force *k_c_*, two types of experiments were carried out. In the first case, tests of longitudinal turning of a tapered surface were carried out, i.e., at a constant value of the feed *f* and variable value of the depth of cut in the range *a_p_* = <0, 1> mm. In the second case, specific cutting force tests were carried out for various shapes of the cross-section of the cutting layer, maintaining a constant value of the cross-section *A_D_*. Figure 1 shows schematically the changing shape of the cross-section of the cutting layer with a constant value. The change in the shape of the cutting layer cross-section results from the selection of the proportions of the dimensions describing the cross-section of the cutting layer, i.e., the feed *f* and the depth of cut *a_p_*.

**Figure 2:**
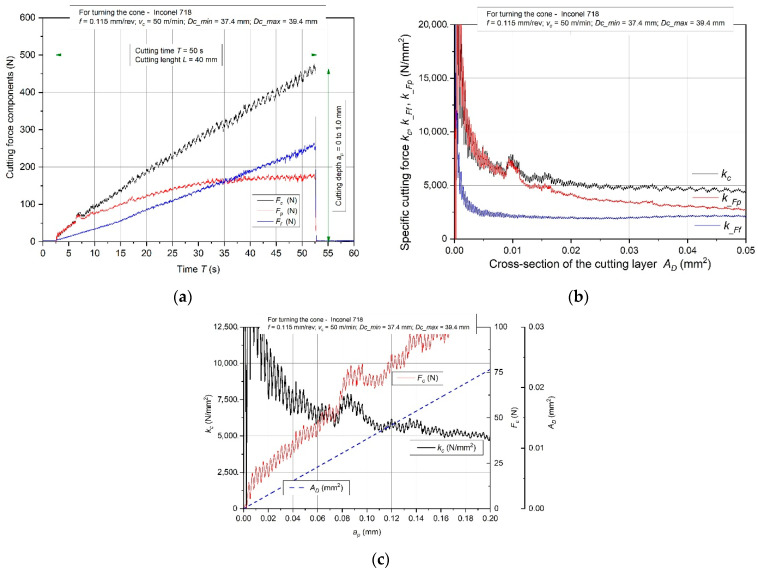
Characteristics of the components *F_c_*, *F_f_*, *F_p_* (**a**) with specific cutting forces (**b**) and specific cutting force *k_c_* during the initial phase of cutting edge work (**c**) for turning the cone made of Inconel 718 alloy.

Figure 2 shows the trends of cutting forces *F_c_*, *F_f_*, *F_p_* and specific cutting forces *k_c_*, *k__Ff_*, *k__Fp_* when turning the conical surface of a shaft made of cast Inconel 718. In this case, the depth of the cut *a_p_*, which changed in time during the turning, resulted in changing the cross-section of the cutting layer. Cutting tests were carried out at a constant feed *f* = 0.115 mm/rev, and the cutting speed *v_c_* = 50 m/min. The cutting force curves were characterized by a constant value increase during machining for all components of the total cutting force (Figure 2a). The specific cutting force values presented in Figure 2b decreased during the cutting process with the increase of the cutting depth *a_p_* and, consequently, with the increase in the cross-section of the cutting layer *A_D_*. It was observed that for turning above the specific value of cutting layer cross-section *A_D_* > 0.04 mm^2^ *k__Ff_* and *k__Fp_*, specific cutting forces are almost constant. Based on the analysis of the trends of total cutting force components, local increases in the values for the main force *F_c_* and the back force *F_o_* were observed, which is shown in detail in Figure 2c. The reason for this phenomenon may be the increasing degree of hardening of the processed material in the surface layer resulting from the performed work of the cutting edge. An additional factor may be the influence of the nose radius of the cutting insert *r_ε_* on the reaching of the minimum cross-section of the cutting layer above which the material decohesion occurs.

**Figure 3:**
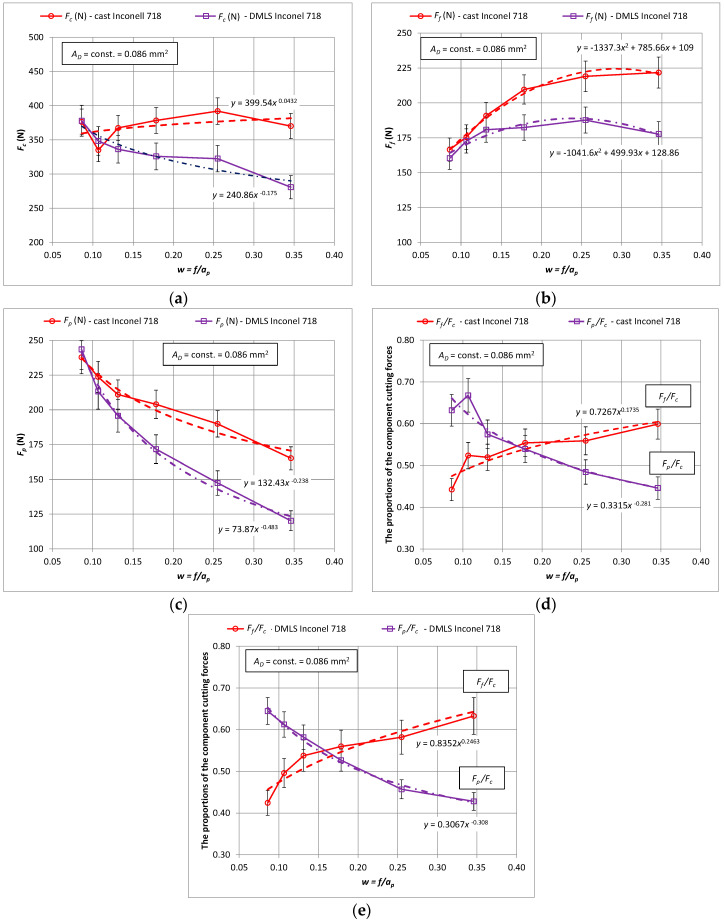
The characteristics of the components for turning with a constant cross-section of the machined layer *A_D_* = 0.8 mm^2^ for the printed and cast Inconel 718 alloy, (**a**) *F_c_* component, (**b**) *F_f_* component, (**c**) *F_p_* component, (**d**) the ratio of the cutting forces *F_f_*/*F_c_* and *F_p_*/*F_c_*–cast material (**e**) the ratio of the cutting forces *F_f_*/*F_c_* and *F_p_*/*F_c_*–3D printed material.

In the next stage of the research, the influence of the shape of cross-section of the cutting layer on the values of the components of the total cutting force and specific cutting force was analyzed. Figure 3 shows the characteristics of the components of the total cutting force *F_c_*, *F_f_*, *F_p_* as a function of the constant cross-section of the cutting layer *A_D_* = 0.8 mm^2^ in 3D printed and cast Inconel 718 alloy.

Based on the analysis of the obtained results, it can be stated that the values of all components of the total cutting force for turning the material obtained by the additive method are lower than for turning the cast one. It was observed that the value differences increase above *w* > 0.15, which describes the ratio of feed *f* to depth of cut *a_p_*. For a ratio of *w* < 0.15, the values of the cutting forces when turning cast and laser sintered material are similar for all components. This means that the greatest differences in the values of the cutting force components are in the case of machining with small depths of cut and high feed values. For example, the values of the main cutting force *F_c_* for turning cast alloy differ by 32% from the value for turning laser sintered material for *w* = 0.35, i.e., the feed *f* = 0.173 mm/rev and the depth of cut *a_p_* = 0.5 mm.

**Figure 4:**
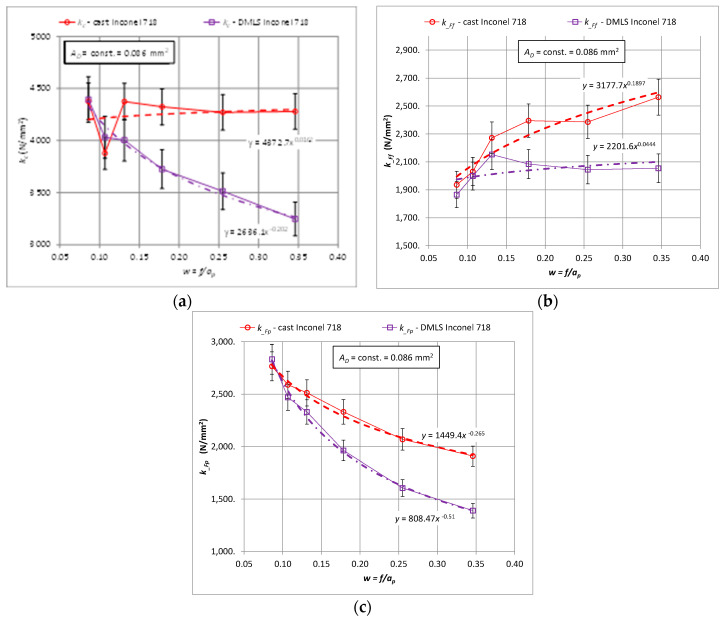
Relationship of specific cutting force for the components *F_c_*, *F_f_*, *F_p_* for turning with a constant cross-section of the cutting layer *A_D_* = 0.8 mm^2^ for the printed and cast Inconel 718 alloy, (**a**) *k_c_*, (**b**) *k__Ff_*, (**c**) *k__Fp_*.

Similar trends as for the characteristics of cutting forces were observed for the corresponding specific cutting forces. Figure 4 shows the characteristics of the specific cutting force *k_c_*, *k__Ff_*, *k__Fp_* for the components of the total cutting force *F_c_*, *F_f_*, *F_p_*, respectively, for turning the 3D printed and cast Inconel 718 alloy.

**Figure 5:**
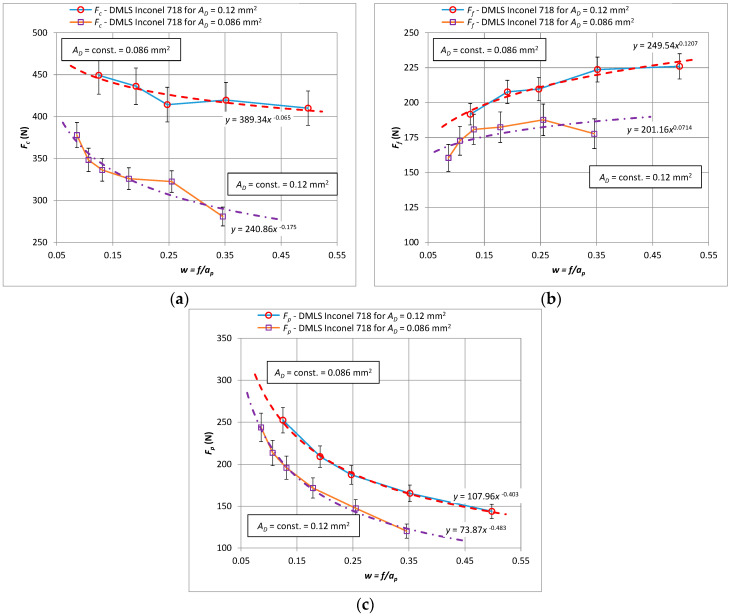
The characteristic of the components for turning with a constant cross-section of the cutting layer *A_D_* = 1.2 mm^2^ for the printed Inconel 718 alloy, (**a**) *F_c_* component, (**b**) *F_f_* component, (**c**) *F_p_* component.

A comparative analysis of the cutting force for the case of increasing values of the cross-section of the cutting layer *A_D_* showed an increase of all components, which are characterized by a constant difference between the values. Figure 5 shows the components of the total cutting force *F_c_*, *F_f_*, *F_p_* as a function of the constant cross-section of the cutting layer *A_D_* = 0.8 mm^2^ and *A_D_* = 1.2 mm^2^ when turning the 3D printed Inconel 718 alloy. A decrease in the *F_c_* and *F_p_* components was observed for increasing values of the w parameter. On the other hand, the values of the feed component *F_f_* increase for each value of the section of the cut layer (Figure 5b).

**Figure 6:**
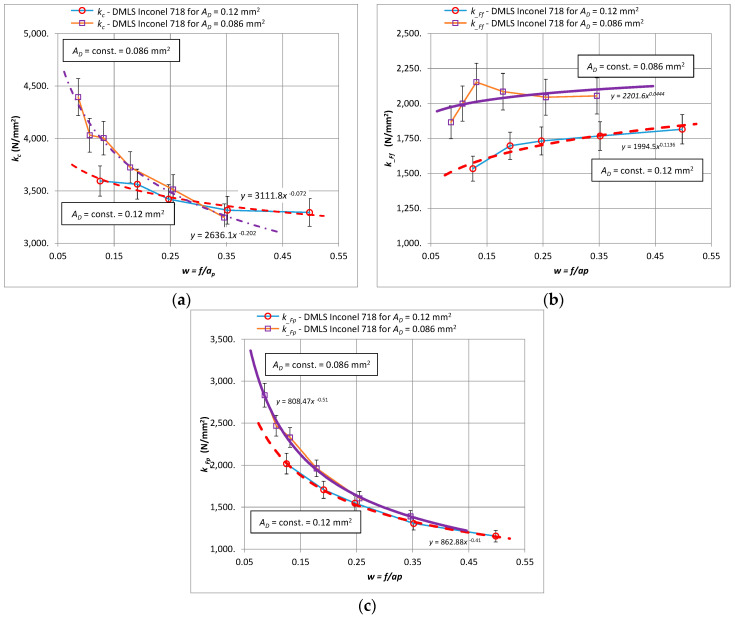
Relationships for specific cutting force components *F_c_*, *F_f_*, *F_p_* for turning with a constant cross-section of the cutting layer *A_D_* = 0.8 mm^2^ and *A_D_* = 1.2 mm^2^ for 3D printed Inconel 718, (**a**) *k_c_*, (**b**) *k__Ff_*, (**c**) *k__Fp_*.

Figure 6 shows the graphs of the specific cutting force *k_c_*, *k__Ff_*, *k__Fp_* for the components of the total cutting force *F_c_*, *F_f_*, *F_p_* respectively, when turning laser-sintered material for two values of the cross-section of the cutting layer, i.e., *A_D_* = 0.8 mm^2^ and *A_D_* = 1.2 mm^2^. The specific cutting force *k_c_* and *k_cp_* decrease with an increase of the parameter *w* (Figure 5a,c) for both analyzed sections. It should be noted that the values of the specific cutting force for the bigger cross-section *A_D_* = 1.2 mm^2^ are lower for *w* parameter range <0.3 than for the cross-section with the lower value. For *w* ≈ 0.3, the values of the specific cutting force *k_c_* reach similar values. Above the value of the parameter *w* ≈ 0.3, the specific cutting force for the cross-section *A_D_* = 1.2 mm^2^ is higher. It seems that by changing the shape of the cross-section of the cutting layer, the turning process can be carried out with reduced specific cutting force values. It is important for the selection of machining allowances and for the implementation of turning, both fine and rough. It should be noted, however, that changing the shape of the surface reduces the volumetric efficiency of the *Q_v_* process, which results in an extension of the total cutting time *T_total_*.

**Figure 7:**
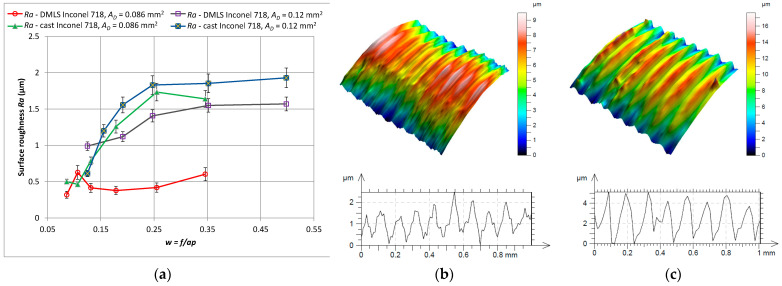
Surface roughness *Ra* relationship for turning with a constant cross-section of the cutting layer *A_D_* = 0.08 mm^2^ and *A_D_* = 0.12 mm^2^ for 3D printed (**a**) and cast Inconel 718 alloy, (**b**) shape and profile-3D printed material, *w* = 0.18, (**c**) shape and profile-cast material, *w* = 0.18.

Figure 7 shows the surface roughness profile *Ra* for turning of the cast and 3D printed Inconel 718 alloy. In all cases, the surface roughness values increased. This is the result of increasing values of the feed *f* in order to maintain a constant cross-section of the cutting layer, i.e., with decreasing values of the depth of cut *a_p_*. It should be noted, however, that when turning laser sintered material, the surface roughness *Ra* was lower than when turning cast material.

**Figure 8:**
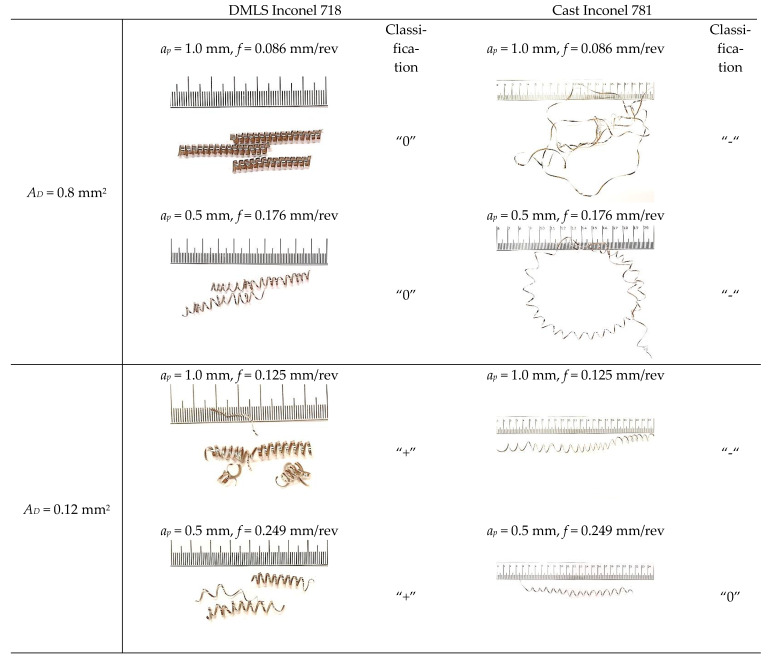
Chips’ photographs obtained for laser sintered and cast alloy turning.

Figure 8 shows examples of photos of chips obtained by turning a laser sintered and cast alloy. In the case of turning the laser sintered alloy, an acceptable chip shape was obtained with a length in the range of *L_ch_* = <30; 60> mm. The following chip classification has been adopted: “-”-unacceptable chips with a length of more than 150 mm; “0”-acceptable chips with a length of 30–150 mm and favorable chips “+” with a length of up to 30 mm. The obtained chips were spiral and continuous. A slight change in the chip shape was observed for increasing the feed *f*. For turning the cast Inconel 718, usually chips were obtained with an unacceptable shape with a length above *L_ch_* > 150 mm. The obtained chips had the form of a long, twisted or spiral ribbon. A change in the chip shape from ribbon to spiral was observed for increasing feed *f* values while maintaining a constant cross-section of the cut layer *A_D_* = 0.08 mm^2^. For a higher value of the cross-section of the cutting layer *A_D_* = 0.12 mm^2^, an advantageous shape of chips was observed when turning the DMLS printed material. In the case of turning cast material, the chips were classified as disadvantageous or acceptable.

## 3. Description of Algorithm for Turning Data Selection and Application

**Figure 9:**
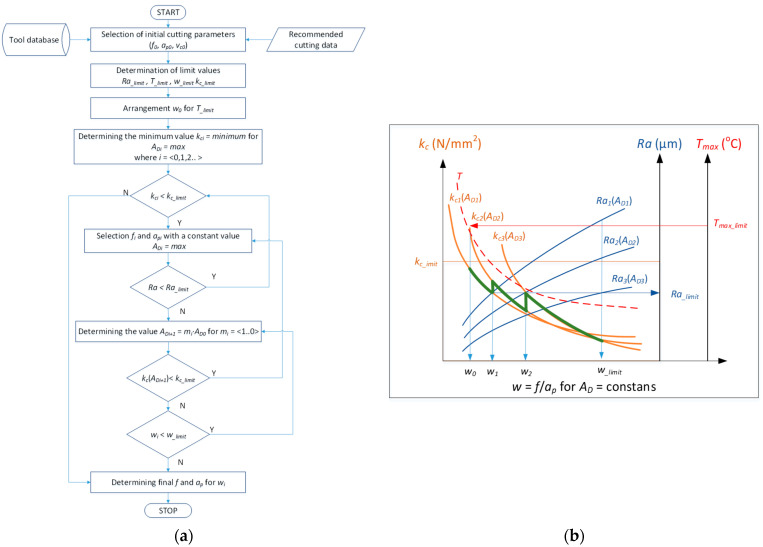
Simplified algorithm of procedure (**a**) for minimizing the value of the actual cutting resistance and the schematic diagram of the method (**b**).

Figure 9 shows a simplified diagram of the procedure algorithm which is used for turning as the method of minimizing the value of the specific cutting resistance *k_c_*. The minimization of the *k_c_* parameter value is performed taking into account the constant value of the area of cross-section of the cutting layer *A_D_*. In turn, the value of the area of cross-section of the cutting layer is determined by selecting the suitable ratio the value of the feed *f* and the cutting depth *a_p_* (schematic presented in Figure 1). The shape of the cross-sectional area of the cutting layer also depends on the corner radius of the cutting insert and the entering angle κr of the applied cutting edge. The method takes into account the optimization constraints as permissible values of specific cutting resistance *k_c_limit_*, surface roughness *Ra*__limit_, *w__limit_*, the coefficient describing the ratio of the feed value and cutting depth and the maximum temperature in the cutting zone, *T_max_limit_*.

The following steps are executed in the procedure algorithm presented in Figure 9a:

1. In the first step, the initial turning conditions are adopted, including the selection of the cutting tool. Based on that, the initial ranges of the values of the cutting parameters (*f*_0_, *a_p_*_0_, *v_c_*_0_) are adopted and the *A_D_*_0_ area of cross-section is determined.
2. In the next step, acceptable values of limiting parameters are adopted, i.e., *k_c_limit_*, *Ra__limit_*, *w_limit_* and *T_max_limit_*.
3. In the next step, the initial value of the parameter *w*_0_ = *f*/*a_p_* is determined for the adopted *T_max_limit_* value.
4. The maximum value of the *A_D_* area of cross-section is determined for which it is possible to obtain the minimum value of the *kc* specific resistance during turning. It also means setting the value of the parameter *w* depending on the feed *f* and the cutting depth *a_p_* in ratio allowing for the maintaining of a constant value of *A_D_* = const. This is done by suitable increasing of the feed value and by decreasing the value of the cutting depth.
5. In the next stages of the procedure algorithm, the actual cutting resistance is checked to see whether it exceeds the allowed values, *k_c_limit_* and *Ra__limit_*. In the case when the condition *Ra* < *Ra__limit_* is not met, another value of *A_D_* is determined, or a correction of the value *A_D_* = *m·A_D_* is adopted. Assuming the value of the correction factor *m* in the range *m* = <1..0> means that a smaller value of the *A_D_* parameter is searched for which a constant value of the parameter w is preserved. At the same time, the condition of exceeding the *k_c_limit_* limit is checked for the determined correction value.
6. As a last step, the condition of exceeding the *w__limit_* value is checked, based on which the final values of the feed and the cutting depth are adopted and the turning is performed.

Figure 9b shows a schematic representation of the implementation steps of the method of minimizing the value of the actual cutting force.

**Figure 10:**
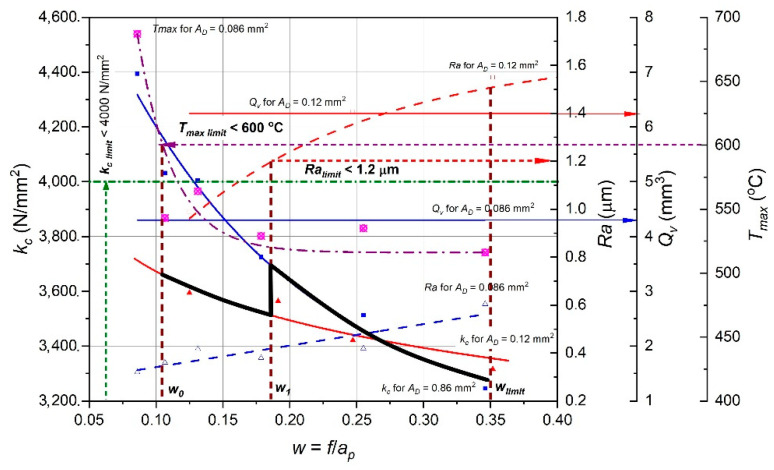
An example of the application of the algorithm for minimizing the value of specific cutting force when turning the Inconel 718 alloy using the DMLS method.

An exemplary algorithm application is presented for turning a shaft made of Inconel 718 with the use of the DMLS method. The following ranges of cutting parameters and acceptable limits were assumed: *ap* = <0.5; 1.0> mm, *f* = <0.086; 0.25> mm/rev, *v_c_* = 50 m/min, *k_c_limit_* = 4000 N/mm^2^, *T_max_limit_* = 600 °C, *w__limit_* = 0.35 and *Ra__limit_* = 1.2 µm. Figure 10 shows the characteristics of the specific cutting force *k_c_*, surface roughness *Ra* and the maximum temperature in the cutting zone *T_max_* as a function of the *w* parameter for two values of the cross-section *A_D_*_1_ = 0.086 mm^2^ and *A_D_*_2_ = 0.12 mm^2^. The figure shows the correct characteristics of specific cutting force, surface roughness and maximum temperature. The adopted limits are marked with horizontal and vertical sections.

− According to the algorithm (Figure 9), for *T_max_limit_* = 600 °C, the initial value of w_0_ = 0.15 was set. For such *w*_0_ value the lowest value of the specific cutting force *k_c_* is for the cross-section *A_D_*_2_ = 0.12 mm^2^.
− For such initial conditions, the lowest value of *kc* may be obtained via selection of the feed *f* (higher *f* value) and depth of the cut *A_D_*_2_ = 0.12 mm^2^. For such assumed initial conditions, the minimization of the *kc* value can be achieved by selecting the feed value *f* (i.e., increasing the *f* value) and the cutting depth *a_p_* (i.e., reducing the ap value) while maintaining the constant value *A_D_*_2_ = const. For the value *w*_1_ = 0.186 the smaller value of the cross-section *A_D_*_1_ = 0.086 mm^2^ should be assumed due to the exceeding of the permissible surface roughness *Ra__limit_* = 1.2 µm at this point.
− Further minimization of the *kc* value is achieved by appropriately (proportional) increasing the value of the feed and at the same time reducing the value of the cut of depth while maintaining a constant value of *A_D_*_1_. The surface roughness characteristics *Ra* for *A_D_*_1_ = 0.086 mm^2^ shows that in this case all surface roughness values are lower than the accepted limit *Ra__limit_* = 1.2 µm. Therefore, the determination of the optimal cutting parameters will result from the adopted limiting value *w__limit_* = 0.35. In this case, the optimal values will be: feed *f* = 0.175 mm/rev and cutting depth *a_p_* = 0.5 mm. However, it should be noted that adopting a smaller value of the cross-section of the cutting layer causes a reduction in the volumetric efficiency of the turning process *Qv* (which results from adopting a constant cutting speed value *v_c_* = 50 m/min). Maintaining the efficiency for the initial cutting data can be achieved by adopting a correspondingly higher cutting speed *v_c_*.

## 4. Conclusions

Based on the obtained results and the conducted analysis, the following conclusions can be made:

− Based on the developed algorithm for turning with a shape of the cross-section of the cutting layer, which changes in time, the machining process can be carried out with reduced specific cutting force values. The algorithm has been experimentally verified for the turning of laser sintered Inconel 718 alloy and can be perceived as a guideline for the mechanical processing of parts obtained by 3D printing.
− Studies have shown that for turning tapered surfaces, the specific cutting force *kc* values decrease with the increase in the cross-section of the cutting layer resulting from the change of the depth of cut *a_p_*.
− The values of all components of the total cutting force for turning the material obtained by the additive method are lower than for turning the cast material. A progressive increase in value differences above the *w* > 0.15 parameter value was observed. The values of the *F_c_* main cutting force for turning a cast alloy vary in the range of 0 to 32% in the analyzed cases. For *w* < 0.15, the values of the cutting forces when turning cast and laser sintered material are similar for all components.
− The surface roughness *Ra* was lower in the machining of the laser sintered material than of the cast material. The surface roughness value *Ra* was within the range of 0.5–2.5 µm.
− A slight influence of the shape of the cross-section of the cut layer on the chip shape was observed. The chip shape was significantly influenced by the value of the *f* feed.

## Figures and Tables

**Table 1:** Mechanical properties of the material Inconel 718 for DMLS and cast parts (at 20 °C).

Material	Tensile Strength (MPa)	Yield Strength Δ(Rp 0.2%)	HRC	Elongation at Break Δ(%)	Density Δ(Cast Part) Δ(g/cm^3^)	Density Δ(DMLS Part) Δ(g/cm^3^)
Inconel 718	1060	780	30	27	8.2	8.15

Rockwell C (HRC) hardness measurement according to EN ISO 6508-1 on polished surface.

**Table 2:** Chemical composition of Inconel 718 (%).

Ni	Cr	Nb	Mo	Ti	Al	Co	Cu	Si	C	Mn
50	18	4.8	3.1	0.95	0.7	<1.0	<0.3	<0.35	<0.08	<0.35

## Data Availability

The data presented in this study are available on request from the corresponding author. The data are not publicly available due to privacy.
